# Dietary Patterns, Alcohol Consumption and Risk of Coronary Heart Disease in Adults: A Meta-Analysis

**DOI:** 10.3390/nu7085300

**Published:** 2015-08-07

**Authors:** Xiao-Yan Zhang, Long Shu, Cai-Juan Si, Xiao-Long Yu, Dan Liao, Wei Gao, Lun Zhang, Pei-Fen Zheng

**Affiliations:** 1 Department of Nutrition, Zhejiang Hospital, Hangzhou 310013, China; E-Mails: shulong19880920@126.com (L.S.); xiaosi_32075001@126.com (C.-J.S.); xly2008hi@163.com (X.-L.Y.); liaodan0203@sina.com (D.L.); gaowei05715133@163.com (W.G.); zhanglun306@163.com (L.Z.); kuaidou09@163.com (P.-F.Z.); 2 Department of Digestion, Zhejiang Hospital, Hangzhou 310013, China

**Keywords:** dietary patterns, coronary heart disease, a meta-analysis

## Abstract

Previous studies reported the potential associations between dietary patterns and the risk of coronary heart disease (CHD) in adulthood, however a consistent perspective has not been established to date. Herein, we carried out this meta-analysis to evaluate the associations between dietary patterns and the risk of CHD. MEDLINE and EBSCO were searched for relevant articles published up to April 2015. A total of 35 articles (reporting 37 original studies) met the inclusion criteria and were included in the present meta-analysis. The decreased risk of CHD was shown for the highest compared with the lowest categories of healthy/prudent dietary patterns (odds ratio (OR) = 0.67; 95% confidence interval (CI): 0.60, 0.75; *p* < 0.00001) and alcohol consumption (OR = 0.68; 95% CI: 0.59, 0.78; *p* < 0.00001). There was evidence of an increased risk of CHD in the highest compared with the lowest categories of the unhealthy/Western-type dietary patterns (OR = 1.45; 95% CI: 1.05, 2.01; *p* = 0.02). The results of this meta-analysis indicate that different dietary patterns may be associated with the risk of CHD.

## 1. Introduction

Although the incidence and mortality of coronary heart disease (CHD) have decreased in the United States and Western Europe since 1970s, it remains the leading cause of death globally, with 7.2 million deaths occurring worldwide every year [1,2,3]. In China, the prevalence of CHD surpassed 80 million by 2010, and every year it causes death in over one million people [4,5]. It is well-known that CHD is considered as a multifactorial chronic disease that may be associated with hypertension, dyslipidemia, impaired glucose tolerance, smoking, genetic factors, and dietary factors [6,7,8].

In the past few decades, many studies particularly focused on diet modification as an important determinant in the development of CHD and found associations between the intakes of individual foods or nutrients and the risk of CHD. However, in reality, people generally do not take nutrients alone but consume meals containing many combinations of foods and nutrients [9]. Consequently, the analysis of dietary patterns has been increasingly used in nutritional epidemiology, taking into account the combined effects of foods, and potentially facilitating nutritional recommendations [10].

Recently, an emerging body of evidence has suggested that there has been considerable attention in epidemiological research on the associations between overall dietary patterns and the risk of CHD [11,12,13,14,15,16,17,18,19,20,21,22,23]. Nevertheless, the results of dietary patterns and CHD risk are inconsistent. Although some studies reported positive associations between Western/unhealthy dietary patterns and the risk of CHD [14,19,22], others showed no significant association [12]. Therefore, we conducted a meta-analysis of studies published up to April 2015, to further identify the potential associations between dietary patterns and the risk of CHD.

## 2. Methods

### 2.1. Literature Search Strategy

An electronic literature search was conducted in MEDLINE and EBSCO to identify human studies written in English or Chinese, published up to April 2015, with the following keywords: dietary pattern, dietary patterns, alcohol drinking, alcohol consumption, cardiovascular disease, ischemic heart disease, myocardial infarction, coronary heart disease and coronary diseases. In addition, we manually searched all references cited in original studies and reviews identified.

### 2.2. Study Included Criteria

Two independent reviewers (L. Shu and X.-Y. Zhang) read the abstracts of the papers retrieved in the initial search to identify studies that examined the associations between dietary patterns and the risk of CHD. When all reviewers agreed, the papers were reviewed against inclusion and exclusion criteria for this meta-analysis. To be eligible, dietary pattern studies had to fulfill the following criteria: (1) The study was published as an original article reporting the relationship of different dietary patterns and the risk of CHD. (2) Food or dietary patterns in studies were examined by principal component analysis (PCA) and/or factor analysis (FA). (3) Odds ratios or hazard ratios, and percentage for CHD (or data can be calculated) had been provided. (4) CHD was diagnosed based on clinical manifestations (including myocardial infarction or angina, or myocardial ischemia, or cardiac failure and arrhythmia, or a death certificate cause of death as CHD), electrocardiogram, and coronary aeteriography. Alcohol studies were included in this meta-analysis if they met the following criteria: (1) the study was published as an original article; (2) the association between alcohol intake and risk of CHD had been reported in studies; (3) the different alcohol consumption categories have been described in studies; and (4) CHD was diagnosed based on clinical manifestations, electrocardiogram, and coronary arteriography.

### 2.3. Data Extraction

Information extracted from each study included authors, geographic region, study design, sample size, the number of CHD, dietary assessment method, identification of dietary patterns, and factors that were adjusted in the including studies.

### 2.4. Definition of “High Intake”

Dietary patterns were identified by principal component analysis or factor analysis. Factor scores for each pattern were categorized into tertiles, quartiles, or quintiles (the lowest category and the highest category represented low and high intake, respectively, to each dietary pattern.) The different forms of alcohol consumption were converted into grams of ethanol per day. Alcohol consumption of >25 g/day for men or >12.5 g/day for women was defined as a high intake of alcohol or heavy alcohol drinking; alcohol consumption of <12.5 g/day for men or <7.5 g/day for women was defined as a low intake of alcohol, and alcohol consumption of >12.5 g/day and <25 g/day for men or >7.5 g/day and <12.5 g/day for women was defined as a moderate alcohol consumption [24].

### 2.5. Quality Assessment

The Newcastle–Ottawa Quality Assessment scale was used for quality assessment [25]. Eight questions were assessed and each satisfactory answer received one point (may receive two points in comparability categories), resulting in a maximum score of nine. Only those studies in which most the questions were deemed satisfactory (*i.e.*, with a score of six or higher) were considered to be of high methodological quality.

### 2.6. Assessment of Heterogeneity

Heterogeneity of the study results was estimated by the chi-squared test. *p* Values less than 0.05 were considered to be significant. In this meta-analysis, a random-effects model was used to account for possible heterogeneity between studies, while a fixed-effects model was adopted in the absence of heterogeneity [26].

### 2.7. Statistical Analysis

Statistical analyses were performed using Review Manager, version 5.0 (Nordic Cochrane Centre, Copenhagen, Denmark) and STATA software, version 12.0 (Stata Corp, College Station, TX, USA). The original studies reported the results of dietary patterns or alcohol consumption in terms of quintiles, quartiles, and tertiles of dietary factor scores and the risk of CHD. We used meta-analysis to evaluate the risk of CHD in the highest *versus* the lowest categories of healthy/prudent, unhealthy/Western-type dietary patterns and alcohol consumption. Random-effect models were used to calculate the pooled odd ratio (OR) for dietary patterns in highest categories compared with lowest categories. Raw data from individual studies were weighted and combined to produce an overall OR. Publication bias was assessed by inspection of the funnel plot and by formal testing for “funnel plot” asymmetry using Begg’s test and Egger’s test [27]. All statistical tests were two-sided and *p* values less than 0.05 were considered significant.

## 3. Results

### 3.1. Overview of Included Studies for the Systematic Meta-Analysis

**Figure 1:**
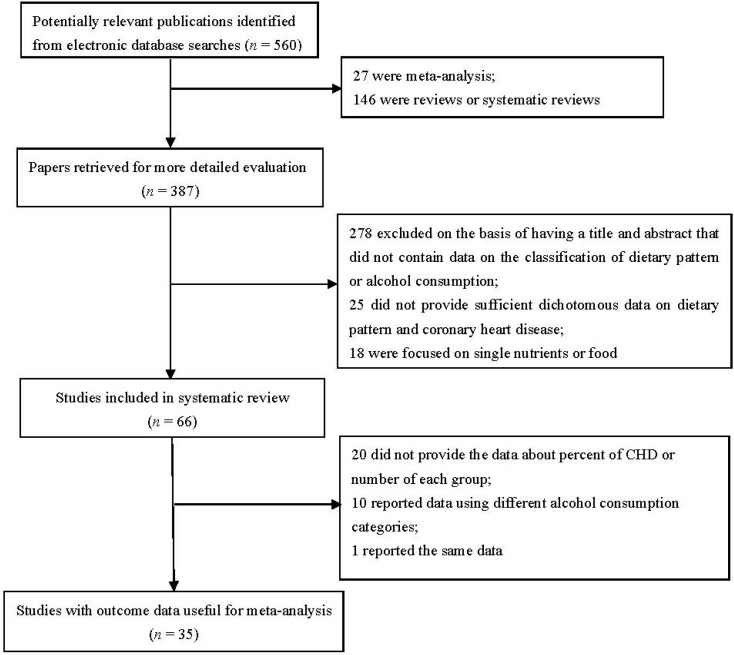
Flow chart of article screening and selection process.

An electronic literature search in the database of MEDLINE and EBSCO identified 560 studies, 525 of which were excluded based on the reasons listed in Figure 1: meta-analysis, reviews or systematic reviews (*n* = 173); title and abstract did not contain the data on classification of dietary pattern or alcohol consumption (*n* = 278); did not provide sufficient dichotomous data on dietary pattern and CHD (*n* = 25); focused on single nutrients or food (*n* = 18); did not provide the data about percent of CHD or number of each group (*n* = 20); reported data using different alcohol consumption categories (*n* = 10); and reported the same data (*n* = 1). At last, 35 articles [8,11,13,14,15,16,17,18,19,20,21,22,23,28,29,30,31,32,33,34,35,36,37,38,39,40,41,42,43,44,45,46,47,48,49] (reporting 37 original studies) met the inclusion criteria and were included in this meta-analysis. Descriptive information of each included study was presented in Table 1.

**Table 1:** Characteristics of 35 studies included in the meta-analysis (1991–2015).

Author and Publication Year	Location	Study Design	Total Number of Subjects	Age (Year)/Sex	Diet Assessment Method	Dietary Patterns Identified	Factors Adjusted for in Analyses
Osler *et.al.*, 2002 [8]	Danish	Cohort	5834	30–60 years both	FFQ	“prudent food” “Western food”	Smoking, exercise, education, BMI and alcohol intake
Fung *et.al.*, 2001 [11]	US	Cohort	69,017	38–63 years women	FFQ	“Prudent” “Western”	Age, period, smoking, BMI, hormone replacement therapy, aspirin use, caloric intake, family history, history of hypertension, multivitamin and vitamin E supplement use, physical activity
Iqbal *et.al.*, 2008 [13]	52 countries	Case-control	5761/10,646	41–70 years both	FFQ	Oriental, Western, prudent	Age, sex, region, education, BMI, physical activity, smoking
Hoffmann *et.al.*, 2004 [14]	Germany	Case-control	200/255	30–80 years women	FFQ	“Western”	Age, cigarette smoking, hormone replacement therapy, hypertension, education level, physical activity level and sport
Stricker *et.al.*, 2012 [15]	Dutch	Cohort	40,011	50–69 years both	FFQ	“Prudent” “Western”	Age, gender, physical activity, smoking status, education, systolic- and diastolic blood pressure and energy intake
Weikert *et.al.*, [16] (CORA) 2005	Germany	Case-control	200/255	30–80 years women	FFQ	“Simplified food pattern”	Age, cigarette smoking, education attainment, BMI, physical activity level, total energy intake, hormone replacement therapy, hypertension, dyslipidemia, and diabetes
Weikert *et.al.*, [16] (EPIC-Potsdam) 2005	Germany	Cohort	26,795	35–65 years both	FFQ	“Simplified food pattern”	Age, cigarette smoking, education attainment, BMI, physical activity level, total energy intake, hormone replacement therapy, hypertension, dyslipidemia, and diabetes
Lipoeto *et.al*., 2004 [17]	Indonesia	Case-control	93/189	men and women	FFQ	“Animal foods”	Age, physical activity and stress level, total energy
Tucker *et.al.*, 2005 [18]	US	Cohort	501	34–80 years men	7-day diet records	“Low SF and high FV”	Age,, total energy intake, BMI, smoking, alcohol use, physical activity score, dietary supplement use
McNaughton *et.al.*, 2009 [19]	UK	Cohort	7314	35–55 years both	FFQ	“Dietary pattern 1” “Dietary pattern 2”	Age, sex, energy misreporting, ethnicity, smoking, alcohol, physical activity, blood pressure and BMI
Fitzgerald *et.al.*, 2012 [20]	US	Cohort	34,827	≥45 years women	FFQ	“DASH”	Randomization status, age, smoking, time-varying postmenopausal status, time-varying hormone therapy use, alcohol intake, energy intake, physical activity, cigarettes per day, BMI, and highest education level
Martínez-Gonzlez *et.al.*, 2011 [21]	Spain	Cohort	13,609	34–43 years both	FFQ	“Mediterranean”	Age, sex, family history of coronary heart disease, total energy intake, physical activity, smoking, BMI, diabetes at baseline, use of aspirin, history of hypertension and history of hypercholesterolemia
Maruyama *et.al.*, 2013 [22]	Japan	Cohort	64,037	40–79 years both	FFQ	“Vegetable” “Animal food” “Dairy product”	Age, BMI, smoking category, walking time, hours of sports, perceived mental stress, total energy intake, history of hypertension and diabetes
Hu *et.al.*, 2000 [23]	US	Cohort	44,874	40–75 years men	FFQ	“Prudent”, “Western”	Age, BMI, time period, cigarette smoking, parental history of myocardial infarction before age 60, multivitamin and vitamin E supplement use, alcohol consumption, history of hypertension, physical activity, total energy intake, and profession
Guallar-Castillón *et.al.*, 2012 [28]	Spain	Cohort	40,757	29–69 years both	Dietary history	“Mediterranean”, “Westernized”	BMI, waist circumference, education, smoking, physical activity at work, physical activity at home, physical activity during leisure time, diabetes, hypertension, hypercholesterolemia, cancer, oral contraceptives, menopausal status, hormone replacement therapy, total energy intake, and stratified by age at recruitment, sex, and center.
Martínez-González *et.al.*, 2002 [29]	Spain	Case-control	171/171	≤80 years both	FFQ	“*Priori* pattern” “Mediterranean” “*Post hoc* pattern”	Smoking, BMI, high blood pressure, high blood cholesterol, diabetes, leisure-time activity, family history of CHD before 60y, aspirin intake and socioeconomic status
Shimazu *et.al.*, 2007 [30]	Japan	Cohort	40,547	40–79 years both	FFQ	“Japanese pattern” “Animal food”	Age, sex, smoking status, walking duration, education, total energy intake, BMI, and history of hypertension
Arriola *et.al.*, 2010 [31]	Spain	Cohort	41,438	29–69 years both	Dietary history questionnaire	Alcohol intake	Centre, smoking status, height and educational level, stratified by age, physical activity index, waist/hip ratio, vitamin E, antithrombotic and antihemorrhagic drugs and energy intake
Beulens *et.al.*, 2007 [32]	US	Cohort	11,711	40–75 years men	FFQ	Alcohol intake	Age, smoking, BMI, physical activity, diabetes, hypercholesterolemia, family history of MI, aspirin use, lipid-lowering therapy, energy intake, and energy-adjusted quintiles of saturated fat, trans fatty acids, sodium, potassium, magnesium, folate, vitamin E, n-3 fatty acids, and dietary fiber.
Bos *et.al.*, 2010 [33]	Dutch	Cohort	10,530	49–70 years women	FFQ	Alcohol intake	Age, smoking, BMI, menopausal status, physical activity, education level, hypercholesterolemia, diabetes, antihypertensive medication, daily energy intake, vitamin E, vitamin C, saturated fat, and fiber intake
Fernández-Jarne *et.al.*, 2003 [34]	Spain	Case-control	171/171	Mean 62 years Both	FFQ	Total alcohol intake	Total energy intake, smoking, BMI, high blood pressure, high blood cholesterol, diabetes, leisure-time physical activity, aspirin use, family history of coronary heart disease, marital status, occupation, study level, olive oil consumption, ratio of monounsaturated to saturated fat, folic acid, and total fiber intake.
Fuchs *et.al.*, 2004 [35]	US	Cohort	14,506	45–64 years men	Dietary questionnaire	Alcohol intake	Age, cigarette-years of smoking, BMI, LDL- and HDL-cholesterol level, waist/hip ratio, educational level, income, sport index, diabetes mellitus, systolic blood pressure, use of antihypertensive medication.
Ikehara *et.al.*, 2009 [36]	Japan	Cohort	19,356	40–69 years men	Self-administered questionnaire	Alcohol consumption	Age, smoking status, body mass index, history of hypertension and diabetes, sports of leisure time, levels of mental stress, presence of flushing and job, marital status, medical checkups and area.
Ikehara *et.al.*, 2008 [37]	Japan	Cohort	83,682	40–79 years both	Self-administered questionnaire	Ethanol intake	Age, smoking status, BMI, history of hypertension and diabetes, frequency of exercise, perceived mental stress, education level, and intake of vegetables, fish, and fruit
Ikehara *et.al.*, 2013 [38]	Japan	Cohort	47,100	40–69 years women	Self-reported questionnaire	Alcohol consumption	Age, smoking status, body mass index, history of diabetes, sports at leisure time, flushing after drinking alcohol, mental stress, menopausal status and area, history of hypertension
Mukamal *et.al.*, 2006 [39]	US	Cohort	38,077	40–75 years men	FFQ	Alcohol consumption	Age, smoking status, body mass index, the presence or absence of diabetes, hypertension, hypercholesterolemia, and a parental history of myocardial infarction, use or nonuse of aspirin, physical activity, intake of energy, and energy-adjusted intake of folate, vitamin E, saturated fat, trans fat, and fiber.
Keil *et.al.*, 1997 [40]	Germany	Cohort	62/1071	45–64 years both	7-day recall	Alcohol intake	Age, smoking, hypertension, BMI
Kono *et.al.*, 1991 [41]	Japan	Case-control	89/271	40–69 years both	Self-administered questionnaire	Alcohol intake	Age, smoking, strenuous exercise, BMI, systemic hypertension, diabetes mellitus, heart disease in parent, job class
Schröder *et.al.*, 2007 [42]	Spain	Case-control	224/1270	25–74 years both	Questionnaire	Alcohol intake	Age, smoking, educational level, leisure-time physical activity, total cholesterol, LDL- and HDL-cholesterol, diabetes, hypercholesterolemia drug treatment, and diagnosed hypertension
Wells *et.al.*, 2004 [43]	New Zealand	Case-control	1381/1892	35–74 years both	Questionnaire	Alcohol intake	Age group, history of CHD, tobacco smoking, leisure-time physical activity, current antihypertensive drug treatment, family history of premature cardiovascular disease, BMI, diabetes, socioeconomic status, income and low education.
Kitamura *et.al.*, 1998 [44]	Japan	Cohort	8476	40–59 years men	Interview	Alcohol intake	Age, serum total cholesterol, cigarette smoking, BMI, left ventricular hypertrophy, and history of diabetes mellitus.
Mukamal *et.al.*, 2006 (*n* = 2) [45]	US	Cohort	4410	≥65 years both	Self-administered questionnaire	Alcohol use	Age, sex, race, education, marital status, smoking, exercise intensity, depression score, frequent aspirin use, BMI, and diabetes mellitus.
Solomon *et.al.*, 2000 [46]	US	Cohort	5103	30–55 years women	FFQ	Alcohol consumption	Age, time period, body mass index, cigarette smoking, parental history of MI before age 60 years, hypertension, hypercholesterolemia, menopausal status/postmenopausal hormone use, aspirin use, multivitamin use, vitamin E supplement use, and physical activity level.
Bazzano *et.al.*, 2009 [47]	China	Cohort	64,597	≥40 years men	Interviewer-administered questionnaire	Alcohol consumption	Age, body-mass index, average systolic blood pressure, physical activity, cigarette smoking, diabetes education, urban or rural residence, and living in North China
Hvidtfeldt *et.al.*, 2010 [48]	Denmark	Cohort	266,986	≥39 years both	FFQ or diet history questionnaire	Alcohol intake	Age, year of baseline questionnaire
Rajpathak *et.al.*, 2010 [49]	US	Cohort	3198	50–79 years women	FFQ	Alcohol intake	Age, race/ethnicity, BMI, smoking, hypertension, high cholesterol, hormone use, regular aspirin use, quintiles of physical activity, duration of DM, intake of saturated fat, PUFA, fiber.

BMI: body mass index; CHD: coronary heart disease; LDL-cholesterol: low-density lipoproteins cholesterol; HDL-cholesterol: high-density lipoproteins cholesterol; WHR: waist hip ratio; FFQ: food frequency questionnaire.

### 3.2. Healthy Dietary Pattern

**Figure 2:**
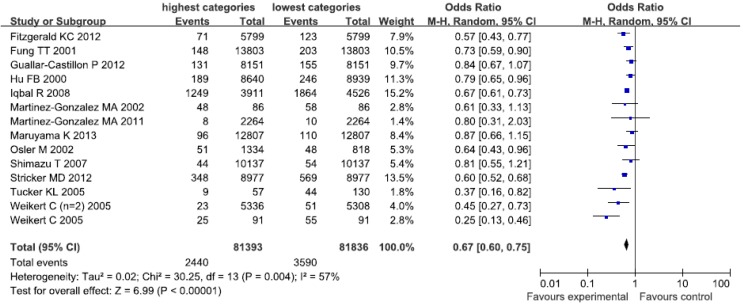
Forest plot of the highest compared with the lowest categories of intake of the healthy/prudent dietary patterns and coronary heart disease (CHD) risk.

The healthy/prudent dietary patterns were characterized by high consumption of vegetables, fruits, whole grains, olive oil, fish, soy, poultry and low fat dairy. The examined studies labeled it as “Prudent” [8,11,13,15,23], “Simplified food” [16], “Low SF and high FV” [18], “Dietary pattern 1” [19], “DASH” [20], “Mediterranean” [21,28,29], “Vegetable and fruit” [22], and “Japanese pattern” [30]. Figure 2 showed an obvious evidence of a decreased risk of CHD in the highest compared with the lowest categories of “healthy/prudent dietary patterns (OR = 0.67; CI: 0.60, 0.75; *p* < 0.00001). A random-effects model was used to assess the data included in our analyses. The heterogeneity was apparent in all the studies (*p* = 0.004; *I*^2^ = 57%).

### 3.3. Western-Type Dietary Pattern

**Figure 3:**
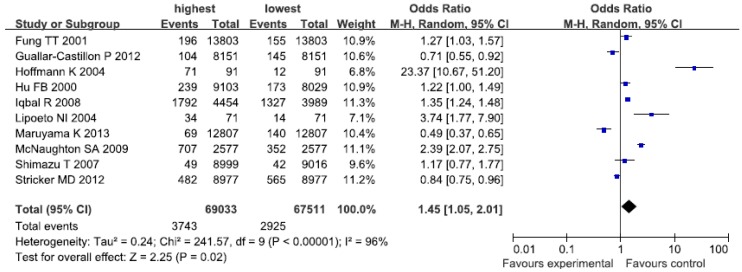
Forest plot of the highest compared with the lowest categories of intake of the unhealthy/Western-type dietary patterns and CHD risk.

The unhealthy/Western-type dietary patterns were characterized by high consumption of red and/or processed meat, refined grains, sweets, high-fat dairy products, butter, potatoes and high-fat gravy, and low intakes of fruits and vegetables. The studies under consideration labeled it as “Western” [8,9,13,14,15,23,28], “Animal foods” [17,22,30] and “Dietary pattern 2” [19]. The association between unhealthy/Western-type dietary patterns and the risk of CHD was shown in Figure 3. There was evidence of an increased risk of CHD in the highest compared with the lowest categories of unhealthy/Western-type dietary patterns (OR = 1.45; CI: 1.05, 2.01; *p* = 0.02) where all studies were combined in the random-effects model. There was significant heterogeneity (*I*^2^ = 96%, *p* < 0.00001).

### 3.4. Alcohol Consumption

**Figure 4:**
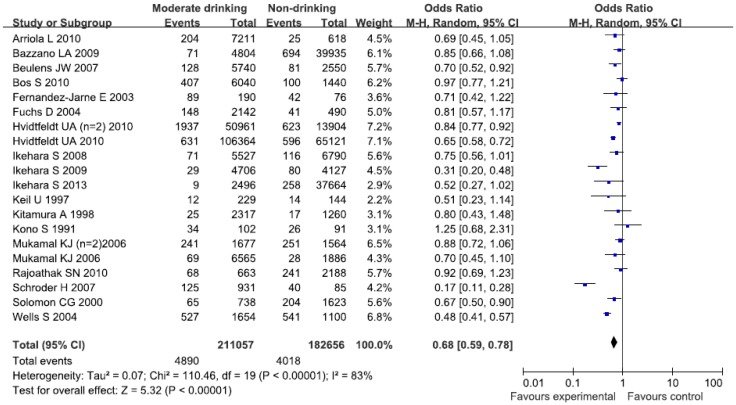
Forest plot of the moderate drinking compared with the non-drinking category of intake the alcohol-drinking pattern and CHD risk.

The alcohol consumption was characterized by moderate intakes of wines, alcohol-containing beers, and white spirits. Nineteen studies (reporting twenty original studies) have identified moderate alcohol consumption. The data from nineteen studies were assessed using the random-effects model, and there was significant heterogeneity (*I*^2^ = 83%, *p* < 0.00001). Figure 4 showed an obvious evidence of a decreased risk of CHD in the moderate drinking compared with non-drinking category intake of the alcohol consumption levels (OR = 0.68; 95% CI: 0.59, 0.78; *p* < 0.00001).

### 3.5. Publication Bias

Inspection of funnel plots did not reveal evidence of asymmetry (Appendix A). Begg’s tests for publication bias were not statistically significant (highest compared with lowest category: healthy/prudent dietary pattern, Begg’s test *p* = 0.285; unhealthy/Western-type dietary pattern, Begg’s test *p* = 0.276; and alcohol consumption, Begg’s test *p* = 0.218).

### 3.6. Quality Assessment

The quality of each study in terms of population and sampling methods, description of exposure and outcomes, and statistical adjustment of data, is summarized in Appendix B. Thirty-one of thirty-five studies received a score of six or higher on the Newcastle–Ottawa Quality assessment scale and were considered to be of high methodological quality [8,11,13,14,15,16,18,19,20,21,23,28,30,31,32,33,34,36,37,38,39,40,41,42,43,44,45,46,47,48,49].

### 3.7. Sensitivity Analysis

When the results were analyzed by removing non-American and non-European studies [17,22,30,36,37,38,41,44,47], the difference in the risk of CHD was shown in Western-type dietary pattern and alcohol consumption (Western-type: OR = 1.53; 95% CI: 1.05, 2.22; *p* < 0.01; alcohol consumption: OR = 0.68; 95% CI :0.51, 0.80; *p* < 0.05). When sample size <10,000 was removed [8,14,16,17,18,19,29,34,40,41,42,43,44,45,46], the difference in the risk of CHD was shown in Western-type dietary pattern (OR = 1.77; 95% CI: 1.08, 2.28; *p* < 0.01). Similarly, when the results were analyzed by removing the studies adjusted for total energy intake [16,17,18,20,21,22,23,28,30,31,32,34,39], the difference in the risk of CHD for those in the highest category compared with the lowest categories of Western-type dietary pattern was detected. Moreover, when the results were analyzed by removing the studies adjusted for sex, no difference in the risk of CHD was found. Finally, when the results were analyzed by removing case-control studies, the difference in the risk of CHD was shown in alcohol consumption (OR = 0.74; 95% CI: 0.66, 0.83; *p* < 0.01). As these variables have a strong effect on association between different dietary patterns and risk of CHD, their differences may partially explain the observed heterogeneity between studies (Table B2).

## 4. Discussion

Limited epidemiological research has reported the associations between dietary patterns and the risk of CHD. To our knowledge, this is the latest meta-analysis evaluating the evidence for dietary patterns and CHD risk. In the present study, we have an update on the earlier systematic review (Li *et al.*, and Hou *et al.*, 2014) [50,51] and further explore the association between moderate alcohol consumption and the risk of CHD. The results of this meta-analysis demonstrate that the healthy/prudent dietary patterns and moderate alcohol consumption may decrease the risk of CHD, whereas unhealthy/Western-type dietary patterns may increase the risk of CHD. Our findings have confirmed the associations between different dietary patterns and the risk of CHD, and provided information that may be translated into public health action for primary prevention of CHD.

In our analyses, the healthy/prudent dietary patterns were associated with a reduced risk of CHD. Our results were in agreement with some previous studies, which reported an inverse association between healthy/prudent dietary patterns and the risk of CHD [11,23]. The apparently protective effect of vegetables, and fruits may be related to high concentration of antioxidant substances (e.g., vitamin C, vitamin E, and other carotenoids compounds). A previous meta-analysis of fruit and vegetables consumption and risk of CHD concluded that high intakes of fruit and vegetables were associated with a decreased risk of CHD [52]. Besides, several studies have also indicated that antioxidants such as vitamin E can slow the rate of oxidation, protecting endothelial cells and vascular [53], thereby reducing the risk of CHD. Furthermore, earlier studies have found that higher intake of folate may decrease the concentration of homocysteine, which may increase the risk of CHD [53,54]. Recently, Pereira *et al.* reported an inverse association between dietary fiber and the risk of CHD [55]. To our knowledge, the possible mechanism is that dietary fiber can modify blood lipid profiles, lower blood pressure, as well as reduce blood glucose concentrations by slowing intestinal absorption [56]. In addition, some clinical and biological investigations have also found that the micro- and macro-constituents of fruit and vegetables may decrease the risk of hypertension, dyslipidemia and diabetes, which are considered as having an important role in the development or progression of CHD [57,58].

The unhealthy/Western-type dietary patterns were associated with an increased risk of CHD in this meta-analysis. Our results were consistent with previous studies [17,19], which indicated that red meat and processed meat consumption were associated with an increased risk of CHD. To our knowledge, there are several plausible explanations for the positive association between Western-type dietary patterns and CHD risk. Firstly, high consumption of red and processed meat is associated with raised total cholesterol, LDL-cholesterol and blood pressure, and greater BMI [59]. As mentioned above, these metabolic changes are related with the risk factors for CHD. Secondly, high temperature commercial cooking or frying, commonly used in preparing processed meats, may generate heterocyclic amines or polycyclic aromatic hydrocarbons, which may increase the risk of CHD and DM [60,61]. Finally, processed meats contain a high content of salt, nitrates and their byproducts (e.g., peroxynitrite), which may be associated with an increased risk of CHD [62].

An inverse association was shown for moderate alcohol consumption and the risk of CHD in our analyses. Previously, a meta-analysis of alcohol consumption and the risk of CHD indicated that moderate alcohol-drinking (≤1.5 drinks/day) was associated with a decreased risk of CHD [63]. In fact, alcohol consumption has been consistently considered an important risk for some chronic diseases, including hypertension and diabetes. Nevertheless, our results found the favorable effect of moderate alcohol consumption on the development of CHD. As we all know, moderate alcohol consumption can raise the concentration of serum high density lipoprotein cholesterol, which may protect against atheroma formation in coronary arteries [64,65]. In addition, it is also associated with the increased vascular wall prostacyclin, thus preventing thrombus formation in coronary arteries [66]. Furthermore, some studies have found that light to moderate alcohol consumption can lower the levels of fasting insulin [67], which is related to the decreased risk of CHD.

### Strengths and Limitations

This meta-analysis has its own strengths and limitations. Firstly, this is the latest meta-analysis reporting the associations between dietary patterns and the risk of CHD. We not only have an update on an earlier systematic review (Li *et al*. and Hou *et al.*, 2014) [50,51], but also further explore the association between moderate alcohol consumption and the risk of CHD. Secondly, the cases of CHD were confirmed based on clinical manifestations, electrocardiogram, and coronary arteriography, avoiding misdiagnosis. Thirdly, no signs of publication bias were evident in the funnel plot, and the statistical test for publication bias was non-significant. However, some limitations should also be mentioned, when interpreting the results of this meta-analysis. Firstly, the principal limitation of this study was the use of potentially biased evidence. Moreover, there was an inconsistent adjustment for potential confounders among the included studies, and we did not exclude the possibility of confounding in this meta-analysis. As a result, the data included in our analyses might suffer from differing degrees of completeness and accuracy. Secondly, ten of thirty-six studies are case-control studies in this meta-analysis. Thus, selection bias is inevitable in our analyses.

## 5. Conclusions

In conclusion, results from this meta-analysis indicate that healthy/prudent dietary patterns and moderate alcohol consumption are associated with a decreased risk of CHD, while unhealthy/Western-type patterns are associated with an increased risk of CHD. In addition, these findings also suggest that a change of diet is essential for the prevention of CHD. Herein, it makes sense to elucidate the potential associations between dietary patterns and the risk of CHD, and provide scientific rationale for formulating dietary guidelines. Further studies are required to confirm the causal associations between dietary patterns and the risk of CHD.

## Figures and Tables

**Figure A1:**
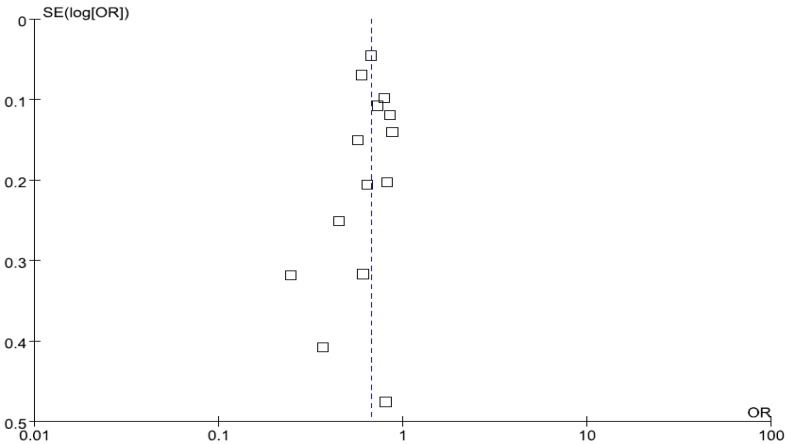
Funnel plots for coronary heart disease(CHD) in the highest compared with the lowest category of intake of the “healthy” dietary pattern in all studies.

**Figure A2:**
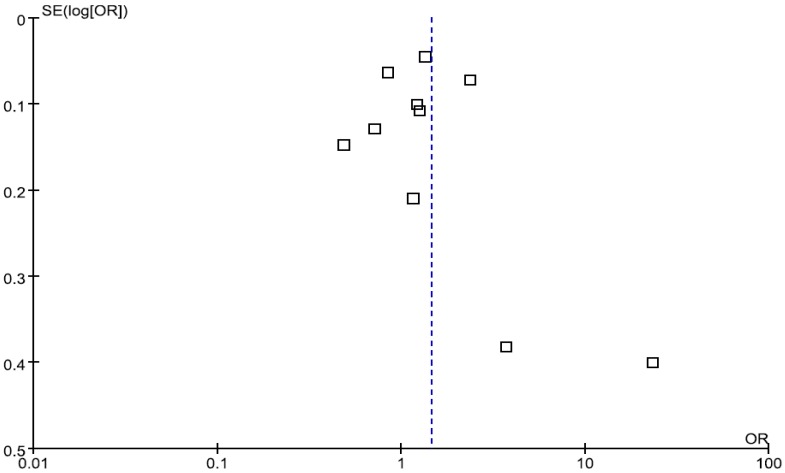
Funnel plots for CHD in the highest compared with the lowest category of intake of the “Western-type” dietary pattern in all studies.

**Figure A3:**
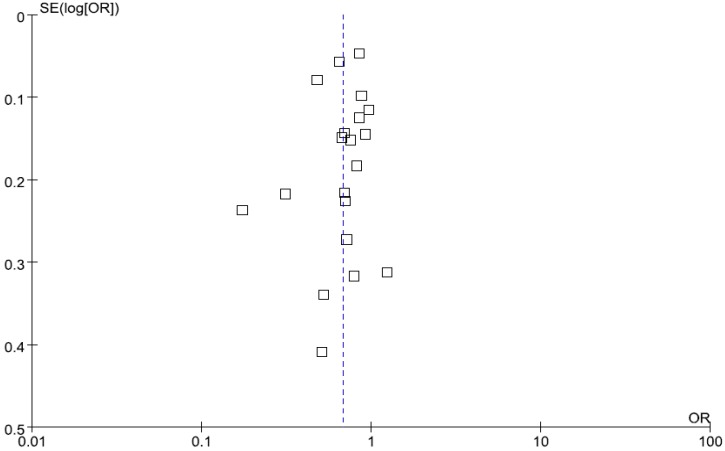
Funnel plots for CHD in the moderate drinking compared with non-drinking category intake of “alcohol consumption” pattern in all studies.

## Appendix B

**Table B1:** Assessment of study quality.

Studies	Selection				Comparability		Outcome			Score
	1	2	3	4	5A	5B	6	7	8	
Cohort										
Fung *et al.*, 2001 [11]		*	*	*	*	*	*	*	*	********
Fitzgerald *et al.*, 2012 [20]	*	*	*	*	*		*	*	*	********
Guallar-Castillón *et al.*, 2012 [28]	*		*	*	*		*	*	*	*******
Hu *et al.*, 2000 [23]	*	*	*	*	*	*	*	*	*	*********
Martínez-Gonzlez *et al.*, 2011 [21]	*	*	*	*	*		*		*	*******
Maruyama *et al.*, 2013 [22]	*		*		*		*	*		*****
Osler *et al.*, 2002 [8]	*	*	*	*	*	*	*	*	*	*********
Shimazu *et al.*, 2007 [30]	*	*	*	*	*		*	*	*	********
Stricker *et al.*, 2012 [15]		*	*	*	*	*	*	*	*	********
Tucker *et al.*, 2005 [18]	*		*	*	*			*	*	******
Weikert *et al.*, 2005 [16]	*	*	*	*	*	*	*	*	*	*********
McNaughton *et al.*, 2009 [19]	*		*	*	*	*	*	*	*	********
Arriola *et al.*, 2010 [31]	*		*	*	*		*	*	*	********
Solomon *et al.*, 2000 [46]		*	*	*	*	*	*	*	*	********
Beulens *et al.*, 2007 [32]	*	*	*	*	*		*	*	*	********
Bos *et al.*, 2010 [33]	*	*	*	*	*	*	*	*	*	*********
Fuchs *et al.*, 2004 [35]	*	*			*		*	*		*****
Ikehara *et al.*, 2008 [37]	*		*	*	*		*	*	*	*******
Ikehara *et al.*, 2009 [36]	*		*	*	*		*	*	*	*******
Ikehara *et al.*, 2013 [38]	*		*	*	*		*	*	*	*******
Keil *et al.*, 1997 [40]	*	*	*	*	*		*	*		*******
Mukamal *et al.*, 2006 [39]	*		*	*	*		*	*		******
Mukamal *et al.*, 2006 (*n* = 2) [45]	*		*	*	*		*	*		******
Bazzano *et al.*, 2009 [47]	*	*	*	*	*		*	*	*	********
Hvidtfeldt *et al.*, 2010 [48]	*		*	*	*		*	*	*	*******
Rajpathak *et al.*, 2010 [49]	*	*		*	*		*	*	*	*******
Kitamura *et al.*, 1998 [44]			*	*	*		*	*	*	******
Case-control										
Iqbal *et al.*, 2008 [13]	*		*	*	*		*	*	*	*******
Martínez-González *et al.*, 2002 [29]	*			*	*		*	*		*****
Weikert *et al.* (CORA) 2005 [16]	*		*	*	*		*	*	*	*******
Lipoeto *et al.*, 2004 [17]	*		*		*		*	*		*****
Hoffmann *et al.*, 2004 [14]	*	*		*	*		*	*	*	*******
Fernández-Jarne *et al.*, 2003 [34]	*	*	*	*	*	*	*	*	*	*********
Kono *et al.*, 1991 [41]	*	*		*	*		*	*	*	*******
Schröder *et al.*, 2007 [42]	*			*	*		*	*		*****
Wells *et al.*, 2004 [43]	*	*	*	*	*	*	*	*		********

* For case-control studies, 1, indicates cases independently validated; 2, cases are representative of population; 3, community controls; 4, controls have no history of blood pressure disease; 5A, study controls for age; 5B, study controls for additional factor (s); 6, ascertainment of exposure by blinded interview or record; 7, same method of ascertainment used for cases and controls; and 8, non response rate the same for cases and controls. For cohort studies, 1 indicates exposed cohort truly representative; 2, non exposed cohort drawn from the same community; 3, ascertainment of exposure; 4, outcome of interest not present at start; 5A, cohorts comparable on basis of age; 5B, cohorts comparable on other factor(s); 6, quality of outcome assessment; 7, follow-up long enough for outcomes to occur; and 8, complete accounting for cohorts.

**Table B2:** Dietary patterns, alcohol consumption and coronary heart disease: sensitivity analysis.

Study Characteristic	Category	Healthy Dietary Pattern (95% CI)	Western-Style Dietary Pattern (95% CI)	Alcohol Consumption (95% CI)
Age	>50	0.65 (0.56, 0.76)	1.31 (0.91, 1.88)	0.72 (0.60, 0.86)
	<50	0.75 (0.63, 0.90)	1.49 (0.90, 2.45)	0.59 (0.45, 0.79)
Sample size	Large (>10,000)	0.71 (0.64, 0.79)	1.21 (0.87, 1.67)	0.72 (0.62, 0.83)
	Small (<10,000)	0.56 (0.37, 0.86)	**1.77 (1.08, 2.88)**	0.64 (0.46, 0.87)
Race	White	0.67 (0.58, 0.77)	**1.53 (1.05, 2.22)**	**0.68 (0.51, 0.80)**
	Asian and Other	0.75 (0.60, 0.92)	1.15 (0.63, 2.11)	0.76 (0.57, 1.06)
Study design	Case-control	0.48 (0.28, 0.84)	4.49 (0.73, 25.75)	0.51 (0.37, 1.07)
	Cohort	0.72 (0.64, 0.81)	1.08 (0.79, 1.48)	**0.74 (0.66, 0.83)**
Total energy	Adjusted	0.67 (0.56, 0.80)	1.05 (0.73, 1.51)	0.78 (0.68, 0.90)
	Unadjusted	0.65 (0.61, 0.70)	**1.99 (1.27, 3.13)**	0.76 (0.55, 0.78)
